# Phosphorylation of Rhoptry Protein RhopH3 Is Critical for Host Cell Invasion by the Malaria Parasite

**DOI:** 10.1128/mBio.00166-20

**Published:** 2020-10-06

**Authors:** Roseleen Ekka, Ankit Gupta, Sonika Bhatnagar, Pawan Malhotra, Pushkar Sharma

**Affiliations:** a Eukaryotic Gene Expression Laboratory, National Institute of Immunology, New Delhi, India; b International Center for Genetic Engineering and Biotechnology, New Delhi, India; Albert Einstein College of Medicine

**Keywords:** phosphorylation, malaria, signaling, invasion, host cell invasion, protein phosphorylation

## Abstract

Merozoites formed after asexual division of the malaria parasite invade the host red blood cells (RBCs), which is critical for initiating malaria infection. The process of invasion involves specialized organelles like micronemes and rhoptries that discharge key proteins involved in interaction with host RBC receptors. RhopH complex comprises at least three proteins, which include RhopH3. RhopH3 is critical for the process of red blood cell (RBC) invasion as well as intraerythrocytic development of human malaria parasite Plasmodium falciparum. It is phosphorylated at serine 804 (S804) in the parasite; however, it is unclear if phosphorylation regulates its function. To address this, a CRISPR-CAS9-based approach was used to mutate S804 to alanine (A) in P. falciparum. Using this phosphomutant (R3_S804A) of RhopH3, we demonstrate that the phosphorylation of S804 is critical for host RBC invasion by the parasite but not for its intraerythrocytic development. Importantly, the phosphorylation of RhopH3 regulates its localization to the rhoptries and discharge from the parasite, which is critical for RBC invasion. We also identified P. falciparum CDPK1 (PfCDPK1) as a possible candidate kinase for RhopH3-S804 phosphorylation and found that it regulates RhopH3 secretion from the parasite. These findings provide novel insights into the role of phosphorylation in rhoptry release and invasion, which is poorly understood.

## Abstract (precis)

Host cell invasion by the malaria parasite is critical for establishing infection in human host and is dependent on discharge of key ligands from organelles like rhoptry and microneme, and these ligands interact with host RBC receptors. In the present study, we demonstrate that phosphorylation of a key rhoptry protein, RhopH3, is critical for host invasion. Phosphorylation regulates its localization to rhoptries and discharge from the parasite.

## INTRODUCTION

*Plasmodium*, the causative pathogen for malaria, claims more than 400,000 lives every year (1). Plasmodium falciparum is responsible for most of the reported mortality caused by malaria. It is the asexual development in human erythrocytes which is mainly responsible for pathogenesis of the disease. After the mosquito bite and release of sporozoites, the parasite develops inside the hepatocytes, resulting in generation of thousands of merozoites. After release into the bloodstream, merozoites invade red blood cells (RBCs) followed by intraerythrocytic development from ring-shaped forms to trophozoites followed by asexual division resulting in ∼30 merozoites per schizont, which egress from the infected RBC (iRBC) to reinvade fresh RBCs.

The process of invasion is a well-orchestrated series of events, which involves merozoite attachment, rearrangement of host RBC membrane, reorientation of the merozoite, tight-junction formation between the apical end of the merozoite and the RBC, and entry into the RBC followed by resealing of the RBC membrane to engulf the parasite. A parasitophorous vacuole (PV) is formed post-invasion within which the parasite resides and develops for the remainder of the time that it spends inside the RBC. After the rupture of the PV membrane (PVM) and the host RBC membrane, the parasite egresses to invade fresh RBCs (2). The process of invasion is tightly regulated and involves discharge of adhesins by specialized apical organelles—micronemes, rhoptries, and dense granules—that serve as ligands for host receptors (3, 4). Rhoptry is composed of two major segments, the neck and the bulb (5), and it releases several adhesins that serve as ligands for receptors on host RBC surface and facilitate the process of invasion. In addition, rhoptry proteins are also speculated to play a role in the formation of the parasitophorous vacuole (PV), and some of these proteins are also involved in host cell remodeling and nutrient uptake (6, 7). There is a limited understanding of the role and regulation of rhoptry bulb proteins. There are two major complexes that reside in rhoptry bulb. One is low-molecular-weight (LMW) complex, comprising RAMA, RAP1, RAP2, and RAP3. RAMA was recently demonstrated to play a key role in invasion (8), and RAP1 seems to be involved in trafficking of RAP2, which is essential for parasite survival (9). The other, high-molecular-weight (HMW) complex, consists of RhopH1/Clag, RhopH2, and RhopH3 (6, 10, 11). Clag exists in at least five forms, 2, 3.1, 3.2, 8, and 9, but each HMW complex can contain only one of the RhopH/Clag isotype (12, 13). The HMW complex is released during invasion and also to the PVM post-entry (14, 15). RhopH1/Clag (16) and interacting proteins RhopH2 (17) and RhopH3 (18, 19) are involved in nutrient acquisition via the new permeability pathways (NPP) that are activated upon parasite entry in the host and are important for intraerythrocytic development. Recently, RhopH3, which is indispensable for the parasite, was shown to regulate both intraerythrocytic development and invasion (18, 19).

Several phosphoproteomic studies have revealed that most rhoptry proteins mentioned above are phosphorylated (20, 21). The role of phosphorylation in the function of rhoptry proteins has remained largely unstudied despite their importance in key parasitic processes. In the present study, we have elucidated the role of RhopH3 phosphorylation in its function. We demonstrate that phosphorylation of S804 is critical for RhopH3 localization to the rhoptries and its discharge from the parasite. While intraerythrocytic development was not dependent on RhopH3 phosphorylation, it plays a major role in the process of host RBC invasion. Recent studies demonstrate that P. falciparum CDPK1 (PfCDPK1) is involved in host RBC invasion by P. falciparum (20). We found that PfCDPK1 interacts with RhopH3 in the parasite and facilitates its phosphorylation.

## RESULTS

### Generation of parasites in which serine 804 of RhopH3 was mutated to alanine.

**FIG 1:**
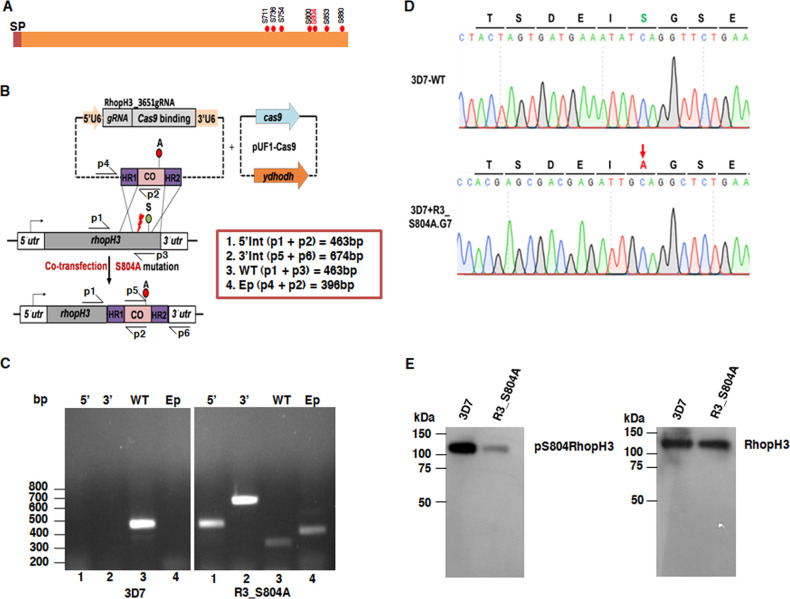
Editing of *RhopH3* to generate a S804A mutant. (A) Schematic illustrating RhopH3 with phosphorylation sites that have been identified by various studies (www.PlasmoDB.org). S804, the subject of the present study, is shown in red font. SP, signal peptide. (B) Illustration depicting the strategy to mutate S804 of RhopH3 to alanine. A PAM site (red arrow) upstream of the S804A codon corresponding to which a gRNA was designed and cloned in pL6 vector is indicated. Two homology arms encompassing the target site, which was codon optimized (CO), were also cloned in the same vector. This construct along with a plasmid construct expressing Cas9 (pUF1-Cas9) was cotransfected in 3D7 parasites. The location of PCR primers used for genotyping is indicated in the figure; the expected sizes of the PCR products that represent 5′ and 3′ integration of the modified locus, the episome, and the wild-type locus are indicated in the inset. (C) Parasites obtained after drug selection were subjected to limited dilution cloning, which was screened by PCR. Genotyping PCR analysis of clone R3_S804A, which was used for subsequent studies, was performed using primers (Table S1) indicated in panel B. PCR performed on genomic DNA isolated from R3_S804A parasites using primers p1+p2 and p5+p6 yielded products corresponding to expected sizes of 463 bp (lane 1) and 674 bp (lane 2), which indicated successful 5′ and 3′ integration at the desired locus, respectively. These products were not observed in the case of 3D7 (WT) parasites. The PCR product obtained using primers p1+p3, which represents the unmodified locus, was observed in the 3D7 but not R3_S804A parasites (left panel). Since clone R3_S804A also retains episome (pL6-R3_S804A) plasmid, a PCR product corresponding to the expected size (396 bp) was obtained upon using primers p4+p2 in clone R3_S804A but not in 3D7 parasites. (D) A PCR product encompassing the modified locus was sequenced. An image of the chromatogram reveals successful editing of the locus of interest (indicated by an arrow) that resulted in the S804-to-A mutation. (E) An antibody was raised against the S804 phosphorylated form of RhopH3, which was used for Western blotting schizont lysates prepared from 3D7 (WT) or R3_S804A mutant. The lysates were also probed with RhopH3_A, which recognizes total RhopH3.

RhopH3 is a component of the HMW rhoptry complex and is involved in the process of RBC invasion (17–19). Various global phosphoproteomics studies on the malaria parasite suggest that it is phosphorylated at several sites (www.plasmodb.org) (20) (Fig. 1A), but the role of phosphorylation in its function has remained unknown. Previous phosphoproteomics studies hinted that protein kinase PfCDPK1 depletion may result in reduced phosphorylation of S804 of RhopH3 in mature schizonts (20) but needed further validation. In order to investigate the role of phosphorylation in RhopH3 function, attempts were made to generate transgenic parasites in which S/T phosphorylation sites of RhopH3 were mutated to nonphosphorylable alanine using a CRISPR-CAS9-based genome editing approach (22) (Fig. 1B). Drug-resistant parasites obtained after transfection of indicated constructs were subjected to limited-dilution cloning. A single clonal population was isolated with the desired modification at the RhopH3 locus as confirmed by genotyping (Fig. 1C). The desired mutation in this clone (R3_S804A) was further confirmed by sequencing which revealed that the codon TCA for serine 804 was mutated to GCA to yield alanine at the 804 position (Fig. 1D). A phosphorylation-site-specific antibody against a phosphopeptide spanning phospho-Serine 804 was generated (see Materials and Methods). While this antibody recognized a band corresponding to RhopH3 in 3D7 parasites, the staining in R3_S804A parasite lysates was significantly reduced as S804 was mutated to alanine, which indicated that this antibody predominantly recognizes S804-phosphorylated RhopH3 (Fig. 1E). The expression levels of RhopH3 protein were almost similar in the two parasite lines.

10.1128/mBio.00166-20.6 TABLE S1 PCR primers used in the study. Download Table S1, PDF file, 0.1 MB. Copyright © 2020 Ekka et al. 2020 Ekka et al. This content is distributed under the terms of the Creative Commons Attribution 4.0 International license.

### Phosphorylation of RhopH3 at S804 is important for invasion.

**FIG 2:**
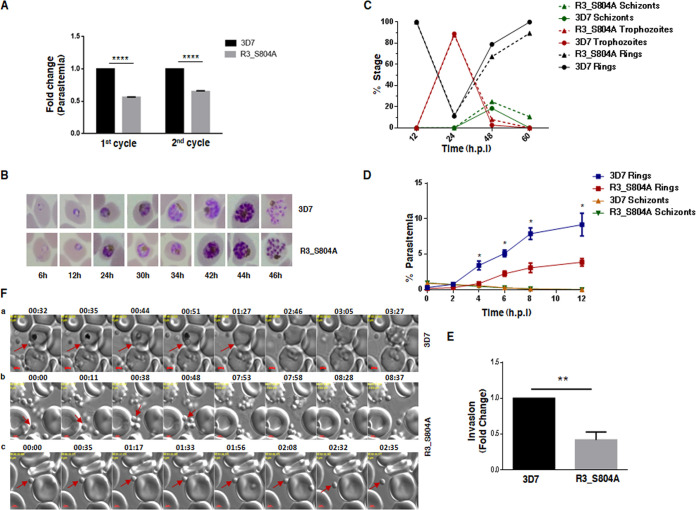
Phosphorylation of RhopH3 at S804 is critical for its role in host RBC invasion. (A) The growth rates of 3D7 and R3_S804A mutants were compared using synchronized parasites. Parasitemia was determined by counting infected erythrocytes from thin blood smears (Fig. S1A), and fold change after completion of first and second cycle at ring stages is provided from three independent replicates (mean ± SD, *n* = 3; ****, *P* < 0.0001, paired *t* test). (B and C) Synchronized ring-stage parasites were plated for the assay, and intraerythrocytic development of 3D7 and R3_S804A was assessed by monitoring Giemsa-stained blood smears at indicated times post-invasion (B). Various parasitic stages were counted at indicated times in both the parasite lines (C). There was no significant difference in intraerythrocytic stages in two parasite lines. A representative of two independent replicates is provided in panel C (another replicate is shown in Fig. S1B). (D) Invasion assay performed with 3D7 or R3_S804A schizonts, which were incubated with fresh erythrocytes. After indicated times, blood smears were prepared (Fig. S1D), and percent schizont- or ring-infected RBCs was determined (mean ± SD, *n* = 4; *, *P* < 0.005, paired *t* test). (E) Fold change in invasion was determined by comparing the ring parasitemia of R3 _S804A parasites with 3D7 at 12 h in invasion assays, which were reported in panel D, from four independent experiments (mean ± SD, *n* = 4; **, *P* < 0.005, paired *t* test). (F) Live imaging was performed on 3D7 and R3_S804A parasites. Selected snapshots from Movie S1 (a) indicate that 3D7 parasites successfully attach to the RBC, deform its membrane, and successfully invade it. R3_S804A parasites attach to the RBC membrane (Movies S2 and S3) for some time and subsequently dissociate from it without invasion (b and c).

First, the intraerythrocytic developments of wild-type (WT) (3D7) and RhopH3_S804A (R3_S804A) mutant parasites were compared by performing growth rate assays. It was evident that the growth of S804A parasites was significantly impaired compared to the wild-type 3D7 counterpart. The major difference in the growth was first apparent after the first cycle as the parasite number was significantly reduced at ∼48 h when most parasites were rings, which persisted in subsequent cycles (Fig. 2A; see also Fig. S1A in the supplemental material). Also, close examination of parasites revealed no major difference in the development of intraerythrocytic stages of R3_S804A parasites (Fig. 2B and C and Fig. S1B). Next, the ability of parasites to egress and invade fresh RBCs was tested. For this purpose, schizonts were incubated with fresh RBCs. There were almost no schizonts left after ∼10 h in both parasite lines, suggesting that egress was largely unaffected in both parasite lines (Fig. 2D). In contrast, ring formation was significantly reduced by more than 50% in the case of R3_S804A parasites as assessed by analyzing Giemsa-stained thin blood smears (Fig. 2D and E and Fig. S1D) or flow cytometry (Fig. S1C). These data strongly indicated that RhopH3-S804 phosphorylation is critical for host RBC invasion.

10.1128/mBio.00166-20.2 FIG S1 (A) The growth rates of 3D7 and R3_S804A mutants were compared using synchronized parasites. Percent parasitemia was determined by counting at least 500 erythrocytes from thin blood smears at indicated times. Mean fold change in parasitemia from three independent replicates at the end of the first two cycles (48 and 96 h) is shown in Fig. 2A. (B) An independent replicate of the experiment described in Fig. 2C. Synchronized ring-stage parasites were plated for the assay, and intraerythrocytic development of 3D7 and R3_S804A was assessed by monitoring Giemsa-stained blood smears at indicated times post-invasion. Various parasitic stages were counted at indicated times in both the parasite lines. There was no significant difference in intraerythrocytic stages in two parasite lines. (C) Invasion assay was performed by incubating 3D7 or R3_S804A schizonts with RBCs as described in Fig. 2D and E. After 12 h, percent parasite-infected RBCs was determined by flow cytometry, and fold change in R3_S804A with respect to 3D7 is provided (mean ± SD, *n* = 3; **, *P* < 0.005, paired *t* test). (D) A representative image of Giemsa-stained thin blood smear from an invasion assay performed with 3D7 or R3_S804A schizonts that were incubated with fresh erythrocytes. Almost no schizonts were left after ∼8 to 10 h, and the number of ring-infected RBCs was significantly less. Download FIG S1, PDF file, 0.1 MB. Copyright © 2020 Ekka et al. 2020 Ekka et al. This content is distributed under the terms of the Creative Commons Attribution 4.0 International license.

10.1128/mBio.00166-20.7 MOVIE S1 Movie for time-lapse microscopy for invasion by 3D7 parasites corresponding to Fig. 2F-a. Download Movie S1, AVI file, 17.9 MB. Copyright © 2020 Ekka et al. 2020 Ekka et al. This content is distributed under the terms of the Creative Commons Attribution 4.0 International license.

10.1128/mBio.00166-20.8 MOVIE S2 Movie for time-lapse microscopy for invasion by R3_S804A parasites corresponding to Fig. 2F-b. Download Movie S2, AVI file, 8.9 MB. Copyright © 2020 Ekka et al. 2020 Ekka et al. This content is distributed under the terms of the Creative Commons Attribution 4.0 International license.

10.1128/mBio.00166-20.9 MOVIE S3 Movie for time-lapse microscopy for invasion by R3_S804A parasites corresponding to Fig. 2F-c. Download Movie S3, AVI file, 12.6 MB. Copyright © 2020 Ekka et al. 2020 Ekka et al. This content is distributed under the terms of the Creative Commons Attribution 4.0 International license.

We further analyzed the invasion process by performing live cell imaging of the two parasite lines. Merozoite invasion of RBC involves several steps that include contact or attachment by the merozoite to the RBC surface, resulting in its deformation followed by parasite entry and recovery of the RBC shape. While 3D7 parasites successfully completed these steps, invaded the RBC, and caused echinocytosis (Fig. 2F-a and Movie S1), R3_S804A either lost contact after initial attachment or was not successful in breaching the RBC membrane (Fig. 2F-b and -c and Movies S2 and S3). These findings suggest that phosphorylation of RhopH3 is important for attachment and/or penetration of RBC.

### Phosphorylation regulates the release of RhopH3.

**FIG 3:**
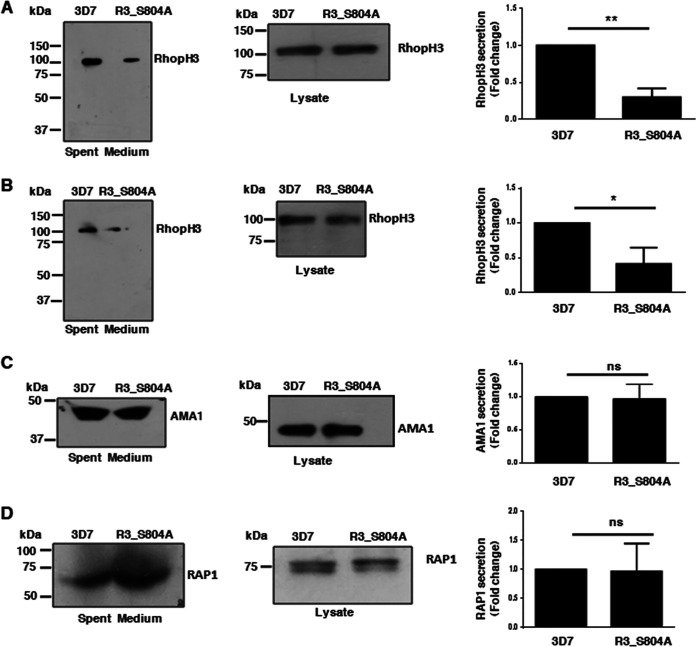
Phosphorylation is important for RhopH3 release from the rhoptries. (A to D) The release of RhopH3 (A and B), AMA1 (C), and RAP1 (D) by parasites was determined by performing Western blotting on spent medium using specific antibodies. Two different antibodies (RhopH3_B [A] and RhopH3_A [B]) were used for detecting RhopH3 (23). The secretion of these proteins in the supernatant was quantitated by densitometry of the Western blot, and the fold change in R3_S804A with respect to 3D7 was determined (A, mean ± SD, *n* = 4; **, *P* < 0.005, paired *t* test; B, mean ± SD, *n* = 4; *, *P* < 0.05, paired *t* test; C, mean ± SD, *n* = 3; not significant [ns], paired *t* test; D, mean ± SD, *n* = 3, paired *t* test). Right panels (A to D), Western blot analyses were also performed on total parasite protein lysates using indicated antibodies.

In order to decipher the mechanism via which RhopH3 phosphorylation regulates invasion, we first tested if its discharge from the parasite is affected. To this end, the level of RhopH3 in spent medium was assessed by immunoblotting with two different antibodies against RhopH3 (23). A significant decrease in RhopH3 in the spent medium was observed in the case of R3_S804A parasites (Fig. 3A and B). However, the total amount of RhopH3 expressed did not show any significant difference, suggesting that RhopH3 expression was unaltered. There was no significant change in the secretion of another rhoptry protein, RAP1, which is part of the LMW complex, as well as microneme protein AMA1 (Fig. 3C and D). These results indicated that S804 phosphorylation is important for the release of RhopH3, which is necessary for the process of invasion (10, 19, 24).

### RhopH3 localization to the rhoptries is regulated by S804 phosphorylation.

**FIG 4:**
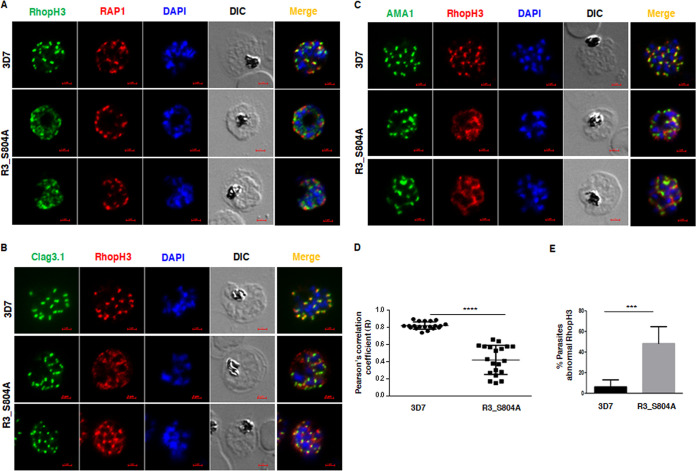
RhopH3 phosphorylation at S804 is important for its localization to the rhoptries. (A to C) IFA was performed on schizont-stage 3D7 or R3_S804A parasites to stain RhopH3 along with RAP1 (A), Clag3.1 (B), and AMA1 (C). While RhopH3 staining was observed in rhoptries as indicated by punctae proximal to RAP1 (A) or colocalized with Clag3.1 (B) in 3D7 parasites, it was significantly diffused in parasite cytoplasm of mutant parasites. The localization of microneme protein AMA1 was unchanged in the mutant parasites (C). (D) Pearson’s correlation was determined, which indicated that RhopH3 colocalizes significantly more with Clag3.1 (C) in 3D7 parasites compared to R3_S804A (mean ± SD, *n* = 20; ****, *P* < 0.0001, paired *t* test). (E) 3D7 or R3_S804A parasites that exhibited loss of typical punctate rhoptry localization were quantitated from IFAs (A to C) (mean ± SD, *n* = 4; ***, *P* < 0.001, paired *t* test).

Since there was a defect in the discharge of the S804A mutant of RhopH3, we investigated if its localization was dependent on the phosphorylation of this site. To this end, immunofluorescence assays (IFAs) were performed on late schizonts/segmenters as RhopH3 is positioned in the rhoptries of merozoites at this stage for the release (8, 19). RhopH3 was localized as puncta adjacent to rhoptry bulb protein RAP1 as expected in 3D7 parasites (Fig. 4A). In sharp contrast, RhopH3 S804A mutant was diffused and did not exhibit exclusive punctae typical of rhoptries in a significant number of parasites (Fig. 4A), which was even more evident after three-dimensional (3D) reconstruction of IFA images (Fig. S4A). RAP1 staining was almost unaltered in these parasites (Fig. 4A and Fig. S4A).

Furthermore, IFA was also performed for Clag3.1, which is part of the RhopH3-containing HMW complex. Clag3.1 exhibited expected punctate rhoptry localization which colocalized with RhopH3 in 3D7 parasites (Fig. 4B). In most R3_S804A parasites, Clag3.1 showed a similar pattern whereas RhopH3 was more diffuse in the cytoplasm and exhibited significant reduction in colocalization with Clag3.1 (Fig. 4B and Fig. S4A), which was also indicated by reduced Pearson’s coefficient in the case of the mutant line (Fig. 4D). More than 50% of R3_S804A parasites exhibited this abnormal localization in which RhopH3 was not found in the rhoptries (Fig. 4E). In a small number of mutant parasites, in some assays, Clag3.1 exhibited dispersed staining (not shown here). Since this was not a consistent observation, it is difficult to conclude if Clag3.1 localization was affected and appeared to be largely unchanged. Moreover, the association of Clag3.1 with RhopH3 complex was almost unchanged as there was no significant difference in Clag3.1 coimmunoprecipitated with RhopH3 (Fig. S4B). The localization of AMA1 to micronemes, which is reflected by punctate apical staining, was also unchanged upon mutation of S804A (Fig. 4C) whereas RhopH3 was significantly mislocalized to the parasite cytoplasm (Fig. 4C).

Collectively, present studies suggest that the phosphorylation of RhopH3 at S804 is critical for its localization to the rhoptries, which is necessary for its discharge during invasion.

### PfCDPK1 may interact with and phosphorylate RhopH3.

**FIG 5:**
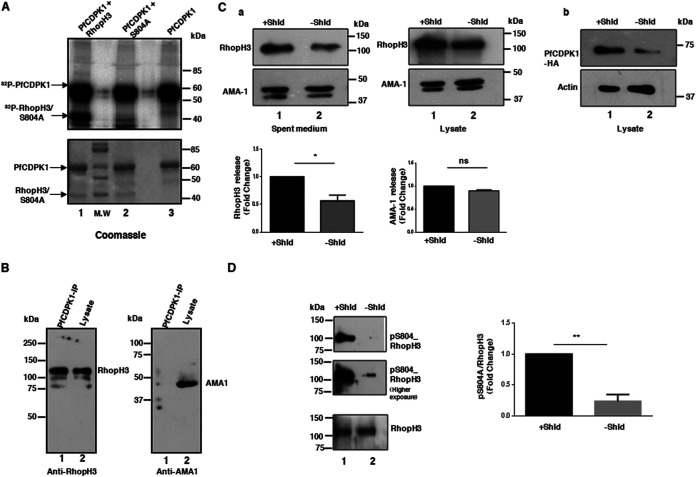
PfCDPK1 mediates RhopH3 phosphorylation at S804 and regulates its release from the parasite. (A) PfCDPK1 kinase assays were performed using a fragment of recombinant RhopH3 or its S804A mutant in an *in vitro* kinase assay using [γ-^32^P]ATP. The assay mix was electrophoresed on an SDS-PAGE gel (lower panel), and phosphate incorporation was observed by phosphorimaging (upper panel). (B) PfCDPK1 was immunoprecipitated from 3D7 parasites using anti-PfCDPK1 antisera, and PfCDPK1-IP or total parasite lysate was used for Western blotting using antibodies against RhopH3 (left) or AMA1 (right). (C) Shld-1 was removed from PfCDPK1-3HA-DD parasites at the ring stage until parasites matured to schizonts. The release of RhopH3 and AMA1 by parasites with or without Shld-1 was determined by performing Western blotting on spent medium with anti-RhopH3_B and anti-AMA1 antibody. RhopH3 secretion but not that of AMA1 was significantly reduced upon Shld-1 removal, which was quantitated by densitometry of the Western blot (lower panel, mean ± SD, *n* = 3; *, *P* < 0.05, paired *t* test; ns, not significant). Parasite lysates were also probed with anti-AMA1 and anti-RhopH3_B (a). Western blotting was also performed with anti-HA to detect PfCDPK1-3HA and anti-actin on schizont lysates (b). (D) Western blotting was performed on lysates of PfCDPK1-3HA-DD schizonts that were cultured in the presence or absence of Shld-1 with an antibody that recognizes RhopH3 phosphorylated at S804 (Fig. 1E) or RhopH3_B antibody to detect total RhopH3. A higher exposure of the pS804 blot is also provided. Right panel, the level of RhopH3 phosphorylation at S804 was quantitated by performing densitometry of the phosphoS804-RhopH3 band, which was normalized with respect to total RhopH3. The fold change in phospho S804-RhopH3 is provided (bottom panel, mean ± SD, *n* = 3; **, *P* < 0.05, paired *t* test).

Having demonstrated that phosphorylation of RhopH3 at S804 is critical for its function in invasion, we wanted to probe the involvement of a parasite kinase. We probed the role of protein kinase PfCDPK1 as reduced phosphorylation of this site was observed upon PfCDPK1 depletion in a data set from a previous phosphoproteomics study (20). This was at best a correlation, which needed further investigation. First, we tested if PfCDPK1 can directly phosphorylate RhopH3 by performing *in vitro* kinase assays. A fragment of RhopH3 (amino acids [aa] 617 to 865), which possesses S804 (23), was used for this purpose. PfCDPK1 phosphorylated RhopH3, and importantly, S804A mutation resulted in almost complete abolishment of phosphorylation, which suggested that S804 can be a target site for PfCDPK1 (Fig. 5A). Next, the association of PfCDPK1 and RhopH3 in the parasite was tested. To this end, PfCDPK1 was immunoprecipitated from 3D7 parasites, and RhopH3 was present in its immunoprecipitate (Fig. 5B), suggesting that these proteins may interact in the parasite, which may facilitate the phosphorylation of RhopH3. In contrast, AMA1 was not present in the PfCDPK1 immunoprecipitation (PfCDPK1-IP) (Fig. 5B, right panel, and Fig. S3B). In addition, reverse IP performed using RhopH3 antisera from PfCDPK1-3HA-DD parasite strain—PfCDPK1 was fused to 3-hemagglutinin (3HA) and FK506 binding protein (FKBP) DD domain (20) in these parasites—also revealed the presence of associated PfCDPK1 (Fig. S3A). Expectedly, no band for HA-tagged PfCDPK1 was observed in IP from 3D7 parasites. These data indicated that PfCDPK1 and RhopH3 may associate in the parasite. We used the phospho-S804 RhopH3 antibody (Fig. 1E) to probe the status of RhopH3 phosphorylation in PfCDPK1-3HA-DD parasites. Since PfCDPK1-3HA is fused to an FKBP-DD domain, the removal of its ligand Shield-1 (Shld-1) results in PfCDPK1 degradation in these parasites (Fig. 5C-b) (20). Western blotting with pS804-RhopH3 antibody revealed a significant reduction in RhopH3 phosphorylation (Fig. 5D and Fig. S2B) in PfCDPK1-depleted parasites. These data suggested that PfCDPK1 may facilitate phosphorylation of this site in the parasite.

10.1128/mBio.00166-20.3 FIG S2 (A) Full version of RhopH3 Western blot analyses that are provided in Fig. 5C that were performed on spent medium and parasite lysate with RhopH3_B antibody. (B) Full version of Western blots shown in Fig. 5D is provided along with the image of Ponceau S-stained membrane. Download FIG S2, PDF file, 0.05 MB. Copyright © 2020 Ekka et al. 2020 Ekka et al. This content is distributed under the terms of the Creative Commons Attribution 4.0 International license.

10.1128/mBio.00166-20.4 FIG S3 (A) RhopH3 was immunoprecipitated from either PfCDPK1-3HA-DD or 3D7 parasites (−ve control) followed by Western blotting with anti-HA antibody to detect 3×HA-tagged PfCDPK1, which was coimmunoprecipitated with RhopH3 from the PfCDPK1-3HA-DD line but not 3D7 parasites (negative control). Data from two independent replicates are provided. An image of Ponceau S-stained membrane is also provided. (B) PfCDPK1 was immunoprecipitated from 3D7 parasites, and IP or total parasite lysate was used for Western blotting with antisera against AMA1. An image of Ponceau S-stained membrane is also provided. Download FIG S3, PDF file, 0.03 MB. Copyright © 2020 Ekka et al. 2020 Ekka et al. This content is distributed under the terms of the Creative Commons Attribution 4.0 International license.

10.1128/mBio.00166-20.5 FIG S4 (A) IFA was performed on schizont-stage 3D7 or R3_S804A parasites to stain RhopH3 (red) along with Clag3.1 or RAP1 (green) followed by microscopy as described in Fig. 4. 3D reconstruction of images revealed that while RhopH3 colocalized with these proteins in 3D7 parasites, it was dispersed in parasite cytoplasm of R3_S804A parasites. (B) An equal amount of 3D7 or R3_S804A schizont lysate was used to immunoprecipitate RhopH3. The IP was further used for Western blot analysis using Clag3.1 antibody. Download FIG S4, PDF file, 0.1 MB. Copyright © 2020 Ekka et al. 2020 Ekka et al. This content is distributed under the terms of the Creative Commons Attribution 4.0 International license.

Since RhopH3 is released by the parasite rhoptries during the process of invasion (23), the effect of PfCDPK1 depletion on its secretion was assessed. To this end, parasite culture supernatant containing parasite secreted material was collected after PfCDPK1 depletion. Immunoblotting with an anti-RhopH3 antibody (23) revealed that the release of RhopH3 was significantly impaired in the case of PfCDPK1-depleted parasites (Fig. 5C and Fig. S2A). In contrast, AMA1, a microneme protein, remained unchanged (Fig. 5C) as reported previously (20). Collectively, these studies suggested that PfCDPK1 may be a candidate kinase that regulates RhopH3 phosphorylation and release from the parasite.

## DISCUSSION

Recently, independent groups demonstrated that RhopH3 depletion results in two major defects in parasite development: (i) abrogation of intraerythrocytic development, primarily maturation of trophozoites, and (ii) defect in invasion of host RBCs (18, 19). In these studies, ∼50% of RhopH3-depleted parasites failed to invade RBCs, which suggested either that residual RhopH3 prevented complete abrogation of invasion or that other alternate invasion pathways (3), which may have compensated for the loss of RhopH3, facilitated this process. Present studies indicate that the mutation of the RhopH3 phosphorylation site S804 to A also reduced invasion by almost ∼40 to 50%. It is clear that phosphorylation of RhopH3 at this site may be critical for this process. However, phosphorylation of RhopH3 at S804 had no apparent role in intraerythrocytic development as R3_S804A parasites did not exhibit any significant change during transition from one developmental form to another.

The possibility of PfCDPK1 phosphorylating RhopH3 was unexpected as it is typically found anchored to the membrane of merozoites and RhopH3 resides in rhoptries (25) and these proteins did not exhibit colocalization under steady-state conditions (not shown here). Previous studies indicated that several HMW and LMW complex rhoptry proteins, including RhopH3, reside in lipid rafts or detergent-resistant membranes (DRMs), which were proposed to traffic them to the rhoptries. Interestingly, PfCDPK1 was also shown to coexist in lipid rafts/DRMs in previous studies (26, 27). Furthermore, an independent study suggested that PfCDPK1 may be present in two different complexes and that one of them is a high-molecular-weight complex which has a phosphorylated form of PfCDPK1 and is localized to the apical end (28). Therefore, we speculate that PfCDPK1 may localize—possibly transiently—to these structures wherein it interacts with and phosphorylates RhopH3. Even though we have provided evidence for PfCDPK1-RhopH3 interaction, which included one-on-one phosphorylation in kinase assays, coimmunoprecipitation in the parasite, and reduced phosphorylation at S804 upon PfCDPK1 depletion, a remote possibility of involvement of another kinase downstream of PfCDPK1 cannot be ruled out. Irrespectively, present studies indicate that RhopH3 phosphorylation at S804 is a PfCDPK1-dependent process. Several phosphoproteomic studies have indicated that RhopH3 is phosphorylated at several other sites as well (Fig. 1A). It will be interesting to see if PfCDPK1 and/or other kinases target these sites and if phosphorylation of other sites also plays a role in RhopH3 function.

Given that mutation of S804A impairs the localization of RhopH3 to the rhoptries, it is reasonable to propose that phosphorylation at this site is critical for its trafficking. A recent study indicated that RhopH3 truncation results in altered localization of other proteins of the complex, like Clag3.1 (19). Since exons 4 to 6 of RhopH3 were lacking in the RhopH3 truncation mutant in these studies, this region may be involved in interaction with Clag3.1. We did not find significant differences in either interaction of Clag3.1 with WT RhopH3 or S804A mutant (Fig. S4B) or its localization in the mutant parasites (Fig. 4B), which was consistent with the presence of exons 4 to 6 in R3_S804A mutant. Therefore, it appears that phosphorylation of RhopH3 at S804 may not affect the HMW complex in schizonts/merozoites. Clearly, present studies demonstrate that phosphorylation of RhopH3 at S804 is important for its localization to the rhoptries as R3_S804A parasites exhibit a significant amount of RhopH3 in the cytoplasm (Fig. 4), which was suggestive of its impaired localization to this organelle. It will be interesting to investigate the mechanism via which phosphorylation of a single residue regulates its localization of RhopH3 to the rhoptries.

It was evident from our studies that ablated rhoptry localization of R3_S804A significantly impaired its release from the parasite (Fig. 3). Several studies hint at a direct role of RhopH3 secretion in invasion: anti-RhopH3 antibodies block the process of invasion (10, 24, 29), and RhopH3 fragments interact with RBC proteins like Band 3 or cyclophilin B, which may serve as its receptors (23, 30). Furthermore, the secreted RhopH3 undergoes cleavage at the C terminus, which may be important for its release from the parasite (31). In the present study, we could not study the RhopH3 cleavage as the antibodies used in our studies recognized mainly the mature fragment. It was only in some experiments that we were able to detect the unprocessed form in the parasite lysate (data not shown). Moreover, we did not see any differences in the expression of mature form in the cell lysate, which possibly rules out a role of phosphorylation in processing of RhopH3. Since the amount of RhopH3 released was significantly reduced in the S804A phosphomutant, the interaction of parasite with RBC receptors is likely to be compromised, preventing efficient invasion. These studies highlight intricate signaling mechanisms that exist in the parasite, which may regulate the function of key parasite ligand proteins like RhopH3 in invasion by phosphorylation.

## MATERIALS AND METHODS

### Reagents and antibodies.

All reagents used in this study were purchased from Sigma-Aldrich. Oligonucleotides were synthesized by Sigma-Aldrich. The following antibodies were purchased from Santa Cruz: mouse anti-β-actin (1:500, C4, sc-47778; Santa Cruz), rabbit anti-HA (1:500, Y-11, sc-805; Santa Cruz), and mouse anti-HA (1:1,000, C12CA5; Roche). Protein A/G Plus agarose beads (sc-2003) were obtained from Santa Cruz Biotechnology. Anti-EBA-175 antibody was obtained from MR4 (1:1,000, MRA-2). Rabbit anti-AMA1 was kindly gifted by Chetan Chitnis, Institut Pasteur, Paris, France (1:1,000, rabbit anti-AMA1) (32). PfCDPK1 antiserum has been used previously (20). Shld-1 was purchased from Cheminpharma, LLC, USA. DSM1 was obtained from MR4 (BEI Resources). WR99210 was obtained from Jacobus Pharmaceuticals. Two rabbit/mouse anti-RhopH3 antibodies indicated here as RhopH3_A and RhopH3_B were generated by Pawan Malhotra (1:2,000). RhopH3 A antibody was raised against fragment A aa 27 to 465, and RhopH3 B antibody was raised against fragment B aa 617 to 865 (23). In most experiments anti-RhopH3 A antibody was used unless stated otherwise. An antibody against PfRhopH3 phosphorylated at S804 was custom generated by Antagene. Inc. (USA). For this purpose, a synthetic peptide with sequence Cys-ASTSDEI(pS)GSEGPST was used to immunize rabbits. Cysteine at the N terminus was added for keyhole limpet hemocyanin (KLH) conjugation. The phosphopeptide antibody was purified using affinity purification, and enzyme-linked immunosorbent assay (ELISA) was performed against the phosphorylated and the nonphosphorylated version of the peptide to determine antibody titer.

### Parasite culture and transfections.

P. falciparum strains were cultured in O^+^ human erythrocytes as described previously (33) in complete RPMI 1640 medium (Gibco) with 0.3% AlbuMAX (Invitrogen) and were maintained under 5% CO_2_, 3% O_2_, and 92% N_2_ at 37°C. Sterile conditions were maintained while handling the cultures outside the laminar hood and incubators to avoid contamination. Transfections were performed using ∼100 μg of purified plasmid DNA constructs in uninfected RBCs followed by the addition of trophozoite-infected parasites. Subsequently, parasites were treated with 1.5 μM DSM-1 and 1.5 nM WR99210. Parasites were synchronized using 5% sorbitol treatment in the ring stage (34). Schizonts were purified by using either Percoll or magnetically activated cell sorting (MACS) columns (LD columns; Miltenyi Biotec, Germany) (35). P. falciparum CDPK1-3HA-DD parasites were cultured in the presence of 2.5 nM WR99210 and 0.25 μM Shld-1, which was used to stabilize the expression of PfCDPK1 in these parasites, as described previously (20).

### Generation of RhopH3_S804A transgenic parasites.

A G-block sequence containing two homology arms encompassing a codon-optimized region containing the S804A mutation was custom synthesized (see Text S1 in the supplemental material) and cloned in pL6-eGFP vector (22) using AflII and SpeI restriction sites. Thereafter, a guide RNA (gRNA) was cloned in the G-block-pL6 construct using BtgZI restriction enzyme and specific oligonucleotides (Table S1). Subsequently, 3D7 parasites were cotransfected with the pL6-RhopH3_S804 and pUF1-Cas9 plasmid and selected with 1.5 μM DSM-1 and 1.5 nM WR99210. Subsequently, drug-selected parasites were subjected to limited-dilution cloning, and a clone, G7 (referred as R3_S804A), was obtained, which exhibited integration at the desired locus and exhibited complete disruption of the wild-type RhopH3. Genotyping of R3_S804A was performed by using sets of PCR primers indicated in Fig. 1B (Table S1). Further, the desired modification was confirmed by PCR amplification of the relevant locus followed by Sanger sequencing.

10.1128/mBio.00166-20.1 TEXT S1 Supplemental materials and methods and supplemental figure legends. Download Text S1, DOC file, 0.04 MB. Copyright © 2020 Ekka et al. 2020 Ekka et al. This content is distributed under the terms of the Creative Commons Attribution 4.0 International license.

### Growth rate and invasion assays.

3D7 and R3_S804A parasites were tightly synchronized using 5% sorbitol (34). Ring-stage parasites were plated at 0.5% to 1% parasitemia in a 6-well plate with 2% hematocrit. Growth rate was monitored periodically by counting parasites from Giemsa-stained thin blood smears. The parasite invasion assay was performed as described previously with some modifications (20, 36). Briefly, parasites were tightly synchronized by sorbitol treatment and were allowed to mature to schizonts, which were purified using MACS columns. Schizonts were washed twice and resuspended in RPMI 1640 medium, and fresh RBCs were added to obtain 2% hematocrit. Invasion assays were set up with ∼1 to 2% schizont parasitemia in a 6-well plate, which was sealed in a zip-lock bag, equilibrated with appropriate gas mix, and kept at 37°C with gentle shaking. Parasite cultures were withdrawn periodically, and thin blood smears were prepared. Parasites were counted from Giemsa-stained smears over several fields to determine the invasion. For assessing invasion using flow cytometry, the parasites were fixed for 30 min with 1% paraformaldehyde (PFA)/0.0075% glutaraldehyde made in ALS (Alsever’s solution; Sigma-Aldrich). Parasites were washed twice with 0.5% bovine serum albumin (BSA)/phosphate-buffered saline (PBS) before staining with Hoechst 33342 (Molecular Probes, Invitrogen, USA) for 20 min. Fluorescence-activated cell sorting (FACS) analysis was performed in a BD Verse machine, and the data were analyzed by using Flow Jo software.

### Secretion of rhoptry and microneme proteins.

The release of microneme and rhoptry proteins was assessed as described previously (20). Briefly, schizonts in culture were allowed to mature and release merozoites and secrete proteins in the extracellular milieu. The culture medium was first centrifuged at 500 × *g* followed by further centrifugation at 3,300 × *g* to pellet down any merozoites present in the supernatant. The supernatant (spent medium) was collected and used for Western blot analysis as described below using antibodies against various proteins of interest.

### Immunoprecipitation and immunoblotting.

Parasites were harvested by 0.05% saponin lysis and were washed with ice-cold PBS and resuspended in lysis buffer (10 mM Tris, pH 7.4, 100 mM sodium chloride, 5 mM EDTA, 1% Triton X-100, 100 μM sodium orthovanadate, 20 μM β-glycerophosphate, 1× protease inhibitor cocktail [Roche], and 10% glycerol). Protein lysates were prepared and clarified by centrifugation at 13,000 rpm at 4°C for 30 min. After separation by SDS-PAGE, lysate proteins (30 to 70 μg) were transferred to a nitrocellulose membrane, which was incubated with relevant primary antibodies, and blots were developed using SuperSignal West Pico or Dura chemiluminescence substrate (Thermo Scientific) following the manufacturer’s instructions.

For immunoprecipitation, 50 to 100 μg parasite lysate was incubated with 5 to 10 μl of anti-PfCDPK1, anti-RhopH3 A/B antiserum, or anti-HA antibody (for IP of PfCDPK1 from PfCDPK1-HA-DD parasites) in complete lysis buffer for 12 h at 4°C. Agarose A/G beads (Santa Cruz Biotechnologies) equilibrated with lysis buffer were added to the IP mix and incubated for another 4 to 5 h at 4°C. The beads containing antibody and bound proteins were washed 5 to 6 times with complete lysis buffer for 5 min followed by centrifugation at 800 rpm for 5 min. Beads were finally resuspended in complete lysis buffer and loaded equally on an SDS-PAGE gel for Western blot analysis. The densitometry of desired bands was performed using Image J software.

### Immunofluorescence assay.

IFA of P. falciparum parasites on thin blood smears was performed as described previously (37). Blood smears were air dried and fixed with ice-cold 1:1 methanol-acetone for 2 min in a Coplin jar and were washed twice with 1× PBS (pH 7.4). Blocking was done using 3% BSA prepared in 1× PBS (pH 7.4) for 2 h at room temperature in a humidified chamber. Subsequently, primary antibody was overlaid on the slide and incubated for 2 h at ambient temperature or 12 h at 4°C in a humid chamber. Smears were washed 4 times in 1× PBS in a Coplin jar for 4 to 5 min each and incubated with Alexa Fluor 488- or 594-conjugated secondary antibodies for 2 h at ambient temperature followed by 4 to 5 washes with 1× PBS of 4 to 5 min each. After drying, mounting was done using Vectashield mounting medium containing 4′,6-diamidino-2-phenylindole (DAPI) (Vector Laboratories Inc.). Subsequently, microscopy was performed as described below.

### Microscopy and image acquisition.

Fluorescently labeled parasites were visualized under an AxioImager ZI inverted fluorescence microscope fitted with an Mrm camera (Carl Zeiss) for microscopic analysis. Images were acquired with a preset exposure time using AxioVision 4.2.8 software, z-stacks were taken for each image, and an appropriate scan was used for illustration in figures. For 3D reconstruction, the z-stack images from the 3D7 and R3_S804A parasites were subjected to the 3D module of the AxioVision 4.2.8 software. The surface interface was used for 3D construction. Pearson’s correlation coefficient was calculated using Zen software (Zeiss).

### Live cell imaging.

After synchronization, parasites were allowed to mature. Subsequently, 3D7 or R3_S804A schizonts were purified using a MACS column as described above and incubated with fresh RBCs at 2% hematocrit in RPMI complete medium followed by plating on cell imaging cover glass (Eppendorf, Germany) coated with 0.5 mg/ml concanavalin A. Live cell imaging of the parasites was performed on a Zeiss Axio Observer microscope. The sample chamber was maintained at 37°C and supplied with a humidified atmosphere under 5% CO_2_.

### Kinase assay.

PfCDPK1 kinase assays were performed as described previously (20). Briefly, recombinant 6×His-PfCDPK1 was incubated with a recombinant fragment of RhopH3 (aa 617 to 865) (23) in a reaction buffer containing 50 mM Tris, pH 7.4, 10 mM magnesium chloride, 1 mM dithiothreitol, 100 μM CaCl_2_, and 100 μM [γ-^32-^P]ATP, 15 μCi/reaction mixture, at 30°C for 45 min. Reaction mix was boiled in SDS-PAGE loading buffer and was electrophoresed, and phosphorylation of proteins was detected by phosphorimaging.

### Statistical analysis.

Statistical analysis was performed using GraphPad Prism software. The mean values from a minimum of 3 biological replicates were used in most cases, and *P* values are indicated in figure legends.
