# Supplementary material for: Phosphorylation of Rhoptry Protein RhopH3 Is Critical for Host Cell Invasion by the Malaria Parasite
Source: mBio. 2020 Oct 6;11(5):e00166-20. doi: 10.1128/mBio.00166-20 (PMC7542355; doi:10.1128/mBio.00166-20)
Supplement: TABLE S1 [file mBio.00166-20-st001.pdf]

Table S1: PCR primers used in the study

Primers used to clone gRNA			
**ID**	**Primer Name**	**Sequence (5' to 3')**	**Remarks**
p7	Rhoph3_3651gRNA-F	TAAGTATATAATATTCATATTAGAAGAAAAAACCAGTTTTAGAGCTA GAA	used for gRNA synthesis, Forward
p8	Rhoph3_3651gRNA-R	TTCTAGCTCTAAAACTGGTTTTTTCTTCTAATATGAATATTATATACTT A	used for gRNA synthesis, Reverse
p9	Rhoph3_3651gRNA_N20-F	CATATTAGAAGAAAAAACCA	used to check integration of gRNA in donor plasmid, Forward
p10	gRNArev-R	TAGGAAATAATAAAAAAGCACC	used to check integration of gRNA in donor plasmid, Reverse
Primers used to check integration			
p1	R3_WT-F	CCGATATTTTCTCCAGAGAAAAAG	used to check for 5’ integration and wild type, Forward
p2	R3_cod_opt-R	TCTTTCGTCTTCTCCTCCAGGATA	used to check for 5’ integration and episomal, Reverse
p3	R3_WT-R	TGATCCTTGGTTTTTTCTTCTAATATG	Used for wild type amplification, Reverse
p4	FCU_K7-F	CTTTAAATTCATGCAAAAATTTACTATAAT	Used to check episomal copy, Forward
p5	R3_cod_opt-F	tATccTgGAgGAgAAgACgAAaGAc	used to check for 3’ integration, Forward
p6	Rhoph3_3utr-R	Gcttcaaatcatgctcacatataag	used to check for 3’ integration, Reverse
Primers used to generate template for sequencing			
	R3_WT-F	CCGATATTTTCTCCAGAGAAAAAG	used to amplify DNA fragment for sequencing, Forward
	R3_HR2-R	ATGcttaaGcgcCAATTCATTTTCAGAAGT	used to amplify DNA fragment for sequencing, Reverse
