# Supplementary figures and images for: Phosphorylation of Rhoptry Protein RhopH3 Is Critical for Host Cell Invasion by the Malaria Parasite
Source: mBio. 2020 Oct 6;11(5):e00166-20. doi: 10.1128/mBio.00166-20 (PMC7542355; doi:10.1128/mBio.00166-20)

**A**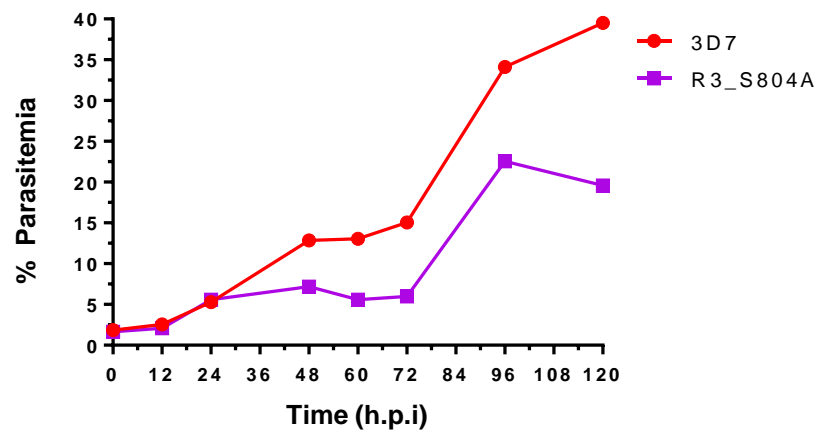**B**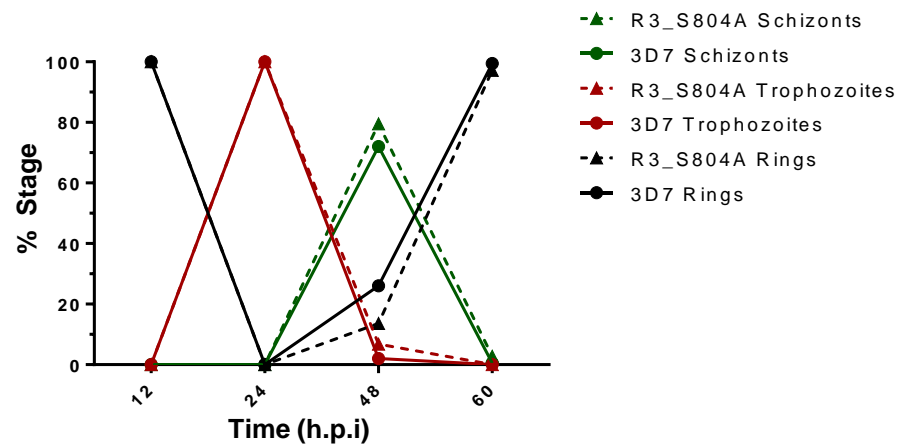**C**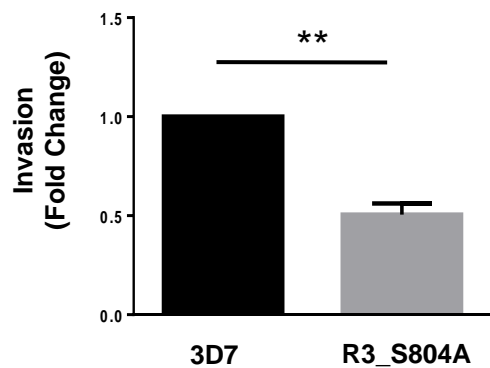**D**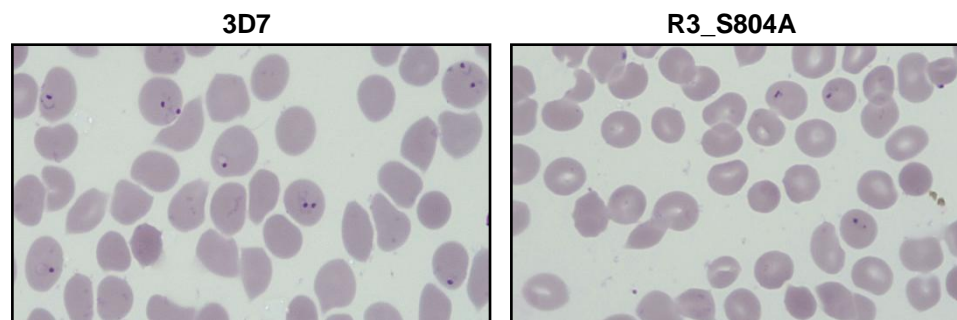

Supplement: FIG S1 [file mBio.00166-20-sf001.pdf]

**A**

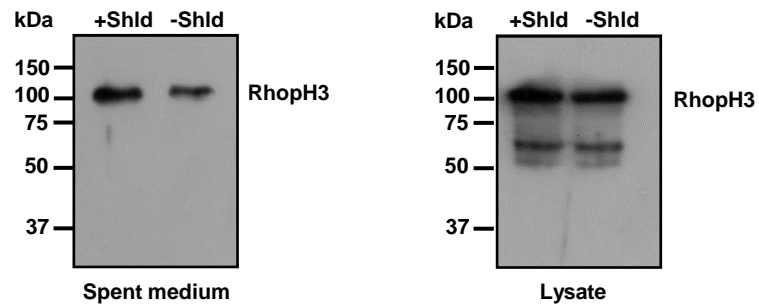

**B**

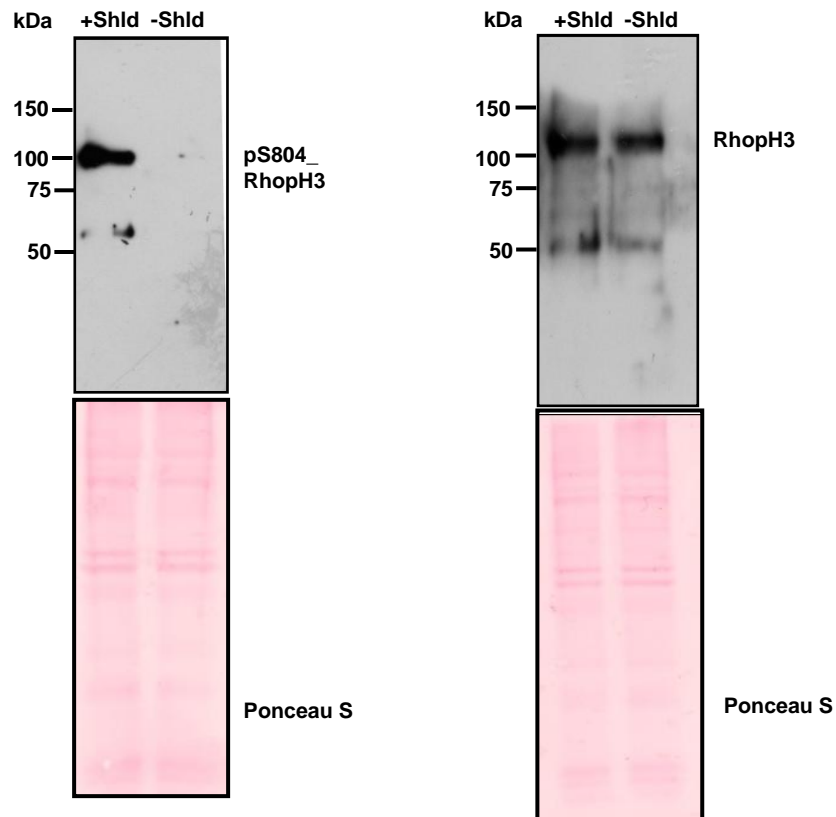

Supplement: FIG S2 [file mBio.00166-20-sf002.pdf]

**A**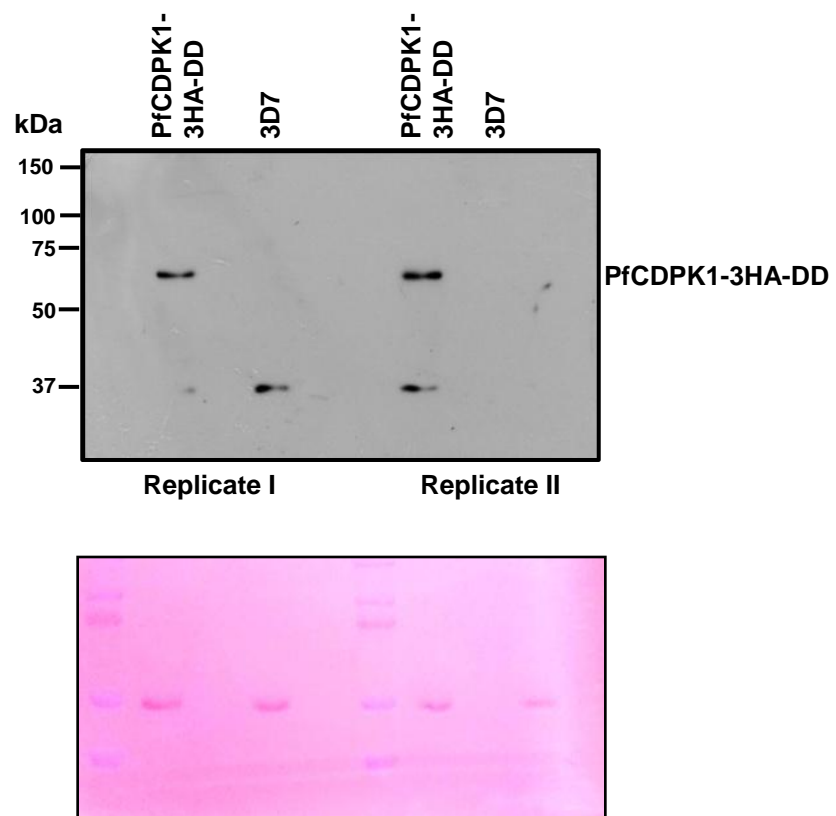**B**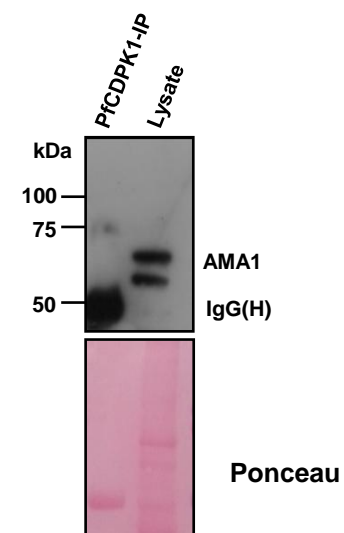

Supplement: FIG S3 [file mBio.00166-20-sf003.pdf]

A

3D7

R3\_S804A

Clag3.1/RhopH3

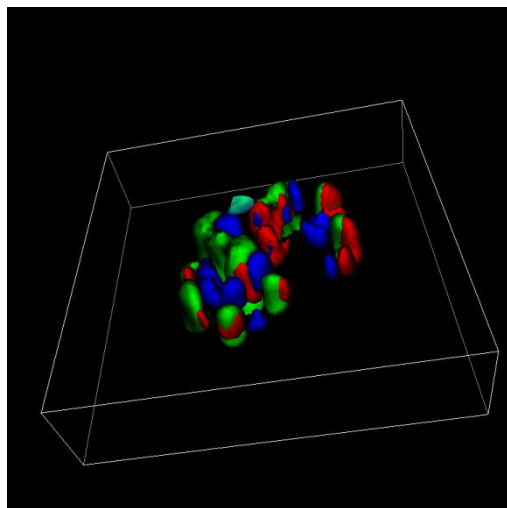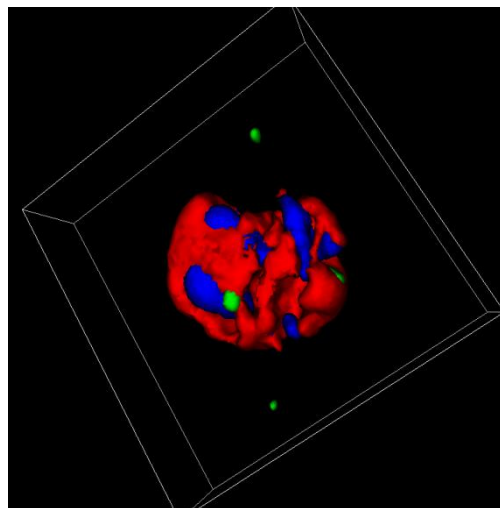

RAP1/ RhopH3

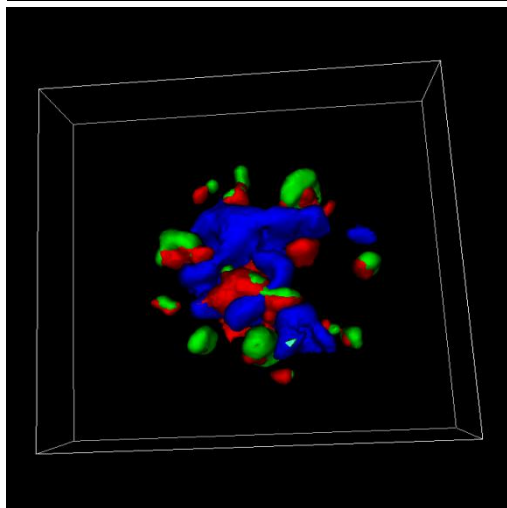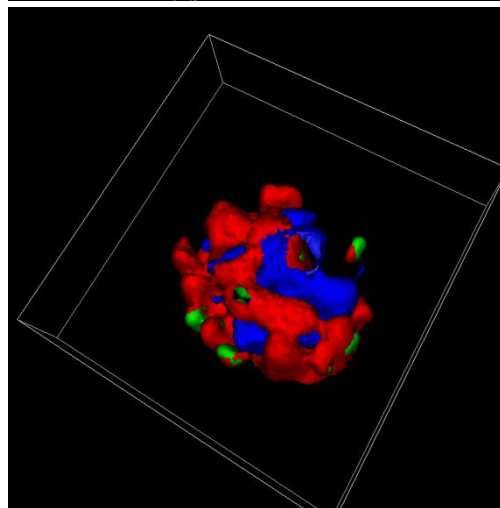

B

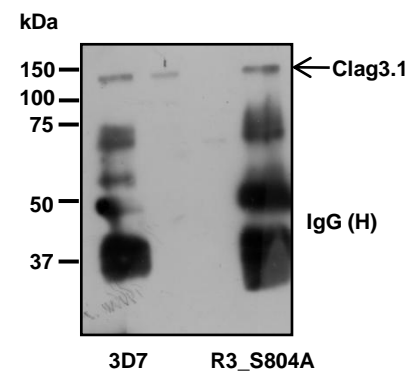

Supplement: FIG S4 [file mBio.00166-20-sf004.pdf]
