# Supplementary material for: Phosphorylation of Rhoptry Protein RhopH3 Is Critical for Host Cell Invasion by the Malaria Parasite
Source: mBio. 2020 Oct 6;11(5):e00166-20. doi: 10.1128/mBio.00166-20 (PMC7542355; doi:10.1128/mBio.00166-20)
Supplement: TEXT S1 [file mBio.00166-20-s0001.doc]

**Supplementary Information**

**Materials**

***Synthesized G-block***

G block with the following sequence was synthesized by Thermo, which was cloned in targeting vector (Figure 1B). Sequence for Homology Region 1 (HR1), Codon Optimized sequence (CO) and Homology Region 2 (HR2 ) (refer to Figure 1B). 15 bp at 5’ and 3’ end corresponding to vector sequence was also synthesized as a part of G block for infusion cloning.

CTTTccgcggggaggactagTTTTTCACCGCCTTATACAACTTCGATAGCTTCATTAAGACCAATGAACAATTAAAGAAGAAGAACTTAGAAGAAATATCAGAAATACCTGTACAATTAGAAACATCTAATGATGGTATTGGATACAGAAAACAAGACGTTCTTTATGAAACTGATAAACCACAAACTATGGATGAAGCTTCATATGAAGAAACTGTAGATGAAGATGCTCACCATGTTAATGAAAAACAACACAGTGCCCACTTCTTAGATGCTATTGCGGAAAAAGAtATccTgGAgGAgAAgACgAAaGAcCAgGAccTgGAgATcGAgcTgTAtAAgTAcATGGGcCCgcTcAAgGAgCAgagTAAgAGcACgAGcGCgGCgTCcACgAGcGAcGAgATtgCAGGcTCTGAAGGTCCATCTACTGAATCTACAAGTACAGGAAATCAAGGTGAAGATAAAACAACAGATAATACATACAAAGAAATGGAAGAATTAGAAGAAGCTGAAGGAACTTCAAATCTTAAAAAAGGTTTAGAATTTTATAAATCTTCTCTAAAACTTGATCAATTAGATAAAGAAAAACCTAAAAAGAAAAAATCTAAAAGAAAAAAAAAGAGAGACAGTTCTAGTGACAGAATATTATTAGAAGAATCTAAAACCTTTACTTCTGAAAATGAATTGgcgCttaagCATTTTGTAAAAAAA

**Supplementary Figure legends:**

**Fig. S1.** **A.** The growth rates of 3D7 and R3-S804A mutants were compared using synchronized parasites. % parasitemia was determined by counting at least 500 erythrocytes from thin blood smears at indicated time. Mean fold change in parasitemia from three independent replicates at the end of first two cycles (48 and 96h) is shown in figure 2A.

**B.** An independent replicate of the experiment described in Fig. 2C. Synchronized ring-stage parasites were plated for the assay and intraerythrocytic development of 3D7 and R3-S804A was assessed by monitoring Giemsa-stained blood smears at indicated time post invasion. Various parasitic stages were counted at indicated time in both the parasite lines. There was no significant difference in intraerythrocytic stages in two parasite lines.

**C.**  Invasion assay was performed by incubating 3D7 or R3_S804A schizonts with RBCs as described in Fig. 2D/E. After 12h, % parasite-infected RBCs was determined by flow cytometry and fold change in R3_S804A with respect to 3D7 is provided (Mean± SD, n=3, ** P<0.005, paired t-test).

**D.** A representative image of Giemsa-stained thin blood smear from an invasion assay performed with 3D7 or R3_S804A schizonts that were incubated with fresh erythrocytes. Almost no Schizonts were left after ~8-10h and the number of ring-infected RBCs were significantly less.

**Fig. S2. A.** Full version of RhopH3 Western blots that are provided in Fig. 5C that were performed on spent medium and parasite lysate with RhopH3_B antibody.

**B.** Full version of Western blots shown in Fig. 5D is provided along with the image of Ponceau S stained membrane.

**Fig. S3.** **A.** RhopH3 was immunoprecipitated either from PfCDPK1-3HA-DD or 3D7 parasites (-ve control) followed by Western blotting with anti-HA antibody to detect 3xHA-tagged PfCDPK1, which was co-immunoprecipitated with RhopH3 from PfCDPK1-3HA-DD line but not 3D7 parasites (negative control). Data from two independent replicates is provided. An image of Ponceau S stained membrane is also provided.

**B.** PfCDPK1 was immunoprecipitated from 3D7 parasites and IP or total parasite lysate was used for Western blotting with antisera against AMA1. An image of Ponceau S stained membrane is also provided.

**Fig. S4. A.** IFA was performed on schizont stage 3D7 or R3_S804A parasites to stain RhopH3 (red) along with Clag3.1 or RAP1 (green) followed by microscopy as described in Figure 4. 3D-reconstruction of images revealed that while RhopH3 co-localizes with these proteins in 3D7 parasites it was dispersed in parasite cytoplasm of R3_S804A parasites.

**B.** Equal amount of 3D7 or R3_S804A schizont lysate was used to immunoprecipitate RhopH3. The IP was further used for Western blot analysis using Clag3.1 antibody.
